# Non-Smoking, Non-Drinking Oral Squamous Cell Carcinoma Is Associated with an Immune-Modulated Clinical Phenotype

**DOI:** 10.3390/cancers18040553

**Published:** 2026-02-08

**Authors:** Marko Tarle, Marina Raguž, Koraljka Hat, Igor Čvrljević, Davor Brajdić, Ivica Lukšić

**Affiliations:** 1 Department of Maxillofacial and Oral Surgery, Dubrava University Hospital, 10000 Zagreb, Croatia; mtarle@kbd.hr (M.T.); korhat@kbd.hr (K.H.); icrvljevic@kbd.hr (I.Č.); dbrajdic@kbd.hr (D.B.); 2 School of Dental Medicine, University of Zagreb, 10000 Zagreb, Croatia; 3 Department of Neurosurgery, Dubrava University Hospital, 10000 Zagreb, Croatia; maraguz@kbd.hr; 4 School of Medicine, Catholic University of Croatia, 10000 Zagreb, Croatia; 5 School of Medicine, University of Zagreb, 10000 Zagreb, Croatia

**Keywords:** oral squamous cell carcinoma, non-smoking non-drinking, immune-mediated carcinogenesis, systemic inflammatory indices, field cancerization

## Abstract

Background: Non-smoking, non-drinking (NSND) oral squamous cell carcinoma (OSCC) is increasingly recognized, yet its clinicopathologic and immune-related correlates remain incompletely defined. Methods: We retrospectively studied 243 surgically treated patients with previously untreated primary OSCC (2011–2020). Patients were classified as NSND or smoking and/or drinking (SD). Immune-modulating conditions and preoperative systemic immune–inflammatory indices (NLR, LMR, SIRI, AISI) were assessed, and overall (OS) and disease-specific survival (DSS) were analyzed. Results: Eighty-five patients (35.0%) were NSND. NSND patients were more often female (58.8% vs. 12.7%) and slightly older (median 54 vs. 50 years). Subsite distribution differed (*p* < 0.001): tongue (52.9%), buccal mucosa (15.3%), and floor of mouth (3.5%) in NSND versus a predominance of floor of mouth tumors in SD (34.8%). NSND tumors showed smaller diameter, lower depth of invasion, less perineural invasion (40.5% vs. 55.1%), and more frequent inflammatory infiltrate (73.8% vs. 60.1%). Immune-modulating conditions were enriched in NSND (67.1% vs. 17.7%; *p* < 0.001; adjusted OR 6.25, 95% CI 3.23–12.11), particularly among NSND patients >50 years (79.2%). NSND patients showed lower NLR (*p* = 0.01), lower SIRI and AISI (*p* < 0.001), and higher LMR (*p* < 0.001). Median OS was 81.2 months; NSND showed a trend toward improved OS (*p* = 0.083) and improved OS after age/sex adjustment (HR 0.64, 95% CI 0.42–0.98), but not after full clinicopathologic adjustment; DSS did not differ (*p* = 0.59). Conclusions: NSND OSCC exhibits a distinct clinicopathologic presentation and is strongly associated with immune-modulating comorbidity and lower tumor-associated systemic inflammatory indices, consistent with an immune-modulated clinical phenotype.

## Simple Summary

Oral cancer is generally associated with smoking and alcohol use, but some patients develop oral squamous cell carcinoma without these exposures. Because these patients do not match the typical risk profile, diagnosis may be delayed. We analyzed 243 treated patients, comparing those who neither smoked nor drank with those who smoked and/or drank. Non-smoking, non-drinking patients were more often women and showed a distinct pattern of tumor sites, with more tumors on the tongue and buccal mucosa and very few on the floor of the mouth. They also more frequently had medical conditions affecting immune function, including autoimmune diseases, especially in patients older than 50 years, and showed inflammatory patterns in blood tests. Overall survival tended to be better, but outcomes were determined by tumor stage and key pathological risk factors. These findings may help clinicians refine risk assessment and follow-up and support research into immune-associated pathways.

## 1. Introduction

Oral squamous cell carcinoma (OSCC) remains a significant global health burden, causing substantial morbidity and mortality worldwide despite advances in surgery, radiotherapy, and systemic therapy [1,2]. According to the most recent global cancer statistics, cancers of the lip and oral cavity account for approximately 390,000 new cases and over 188,000 deaths annually, making OSCC the most common malignancy of the head and neck region and responsible for about 40% of all head and neck cancers globally [3]. Prognosis is largely determined by established clinicopathologic factors, including tumor stage, nodal disease, and depth of invasion (DOI), which are incorporated into the 8th edition of the AJCC/UICC TNM classification for oral cavity cancer. In most settings, tobacco and alcohol remain the dominant and synergistic etiologic drivers of OSCC; consequently, classical exposure-associated cohorts are characterized by a marked male predominance, frequent floor-of-mouth involvement, and carcinogen-related field cancerization [4].

However, a clinically relevant proportion of OSCC arises in patients who report neither smoking nor alcohol consumption. This non-smoking, non-drinking (NSND) subgroup has been described for decades, and contemporary institutional and population-based series consistently report that NSND patients account for approximately 20–35% of OSCC cases, depending on cohort characteristics and exposure definitions [5,6]. NSND OSCC has repeatedly been shown to be more common among women and to exhibit a distinct anatomic distribution, most notably with a predominance of oral tongue tumors, supporting the concept that NSND OSCC represents a distinct clinical phenotype rather than a residual category defined solely by the absence of classical exposures [7]. This emerging epidemiologic pattern is clinically important, as etiologic heterogeneity may influence tumor presentation, comorbidity profiles, surveillance strategies, and potentially therapeutic response [8]. The biological basis of NSND OSCC remains incompletely understood and is likely multifactorial. Unlike oropharyngeal squamous cell carcinoma (OPSCC), where human papillomavirus (HPV)-driven carcinogenesis is well established, the role of HPV in oral cavity cancer is inconsistent and generally limited, prompting investigation of alternative pathogenic pathways in NSND disease [9,10]. Among these, chronic inflammation and immune dysregulation have emerged as compelling candidates. In the oral cavity, immune-mediated and inflammatory conditions can lead to sustained epithelial injury–repair cycles, altered immune surveillance, and chronic cytokine exposure, processes that may contribute to malignant transformation and field effects [11,12]. Oral potentially malignant disorders (OPMD), particularly oral lichen planus, have therefore gained renewed attention in NSND cohorts, with recent systematic reviews supporting a low but measurable malignant transformation risk when strict diagnostic criteria are applied [13,14].

Increasing evidence suggests that NSND OSCC may have an immune-involved tumor microenvironment. Studies of NSND patients have reported differences in tumor-infiltrating lymphocytes and immune checkpoint-related pathways, indicating that the host immune background may influence tumor biology in this subgroup [15,16]. At the systemic level, blood-based immune–inflammatory indices, such as the neutrophil-to-lymphocyte ratio (NLR), lymphocyte-to-monocyte ratio (LMR), systemic inflammation response index (SIRI), and aggregate index of systemic inflammation (AISI) have been associated with prognosis in OSCC and other solid tumors [17,18]. However, these markers have rarely been integrated with clinically defined immune-related comorbidity profiles to characterize etiologic phenotypes such as NSND OSCC. Another important aspect of OSCC biology is field cancerization, reflected in the risk of recurrence, second primary tumors, and multifocal disease [19,20]. While traditionally attributed to chronic carcinogen exposure, immune-mediated or inflammation-driven field effects represent a plausible alternative framework in NSND OSCC, particularly in older patients, in whom immunosenescence and cumulative immune dysregulation may increase long-term mucosal vulnerability.

Despite these observations, important gaps remain. Many studies of NSND OSCC focus on demographic or anatomic features, while fewer integrate immune-modulating comorbidity, systemic inflammatory markers, and clinicopathologic characteristics within a single analytical framework. Moreover, survival outcomes in NSND OSCC remain heterogeneous across the literature, likely reflecting differences in phenotype definitions, subsite composition, and adjustment for established prognostic factors [5,7,8,17,18]. Therefore, in this retrospective cohort of surgically treated OSCC patients, we aimed to characterize NSND OSCC as a clinicopathologic and immune-associated phenotype. We compared NSND and SD patients with respect to tumor subsite distribution, established pathological risk features, immune-modulating comorbidities (including autoimmune diseases), systemic immune–inflammatory indices, and survival outcomes. By integrating immune-related comorbidity with pathology and systemic biomarkers, this study seeks to refine the clinical definition of NSND OSCC and provide a framework for future mechanistic research and risk-adapted surveillance strategies.

## 2. Materials and Methods

### 2.1. Study Design and Data Sources

This retrospective observational cohort study included patients with histopathologically confirmed OSCC treated at the Department of Maxillofacial Surgery, Dubrava University Hospital, between January 2011 and December 2020. Clinical, surgical, and pathological data were retrieved from institutional electronic medical records, operative reports, and pathology databases. The study was designed and reported in accordance with the STROBE (Strengthening the Reporting of Observational Studies in Epidemiology) guidelines.

### 2.2. Study Population

Consecutive adult patients with previously untreated, primary OSCC who underwent surgical treatment with curative intent, with or without neck dissection, were eligible for inclusion. Patients were included if smoking status, alcohol consumption, and follow-up data were available. Patients with a history of prior treatment for head and neck malignancy, including surgery, radiotherapy, or systemic therapy to the head and neck region, were excluded. Patients with non-squamous histology, non–oral cavity primary sites, recurrent disease, or missing key exposure or outcome data were also excluded. A total of 243 patients met the inclusion criteria and were included in the final analysis. A STROBE-style flow diagram summarizing cohort assembly and analytic datasets (including evaluable denominators for recurrence and laboratory-derived indices) is provided in Supplementary Figure S1.

### 2.3. Definition of Exposure: NSND and SD Phenotypes

Smoking and alcohol consumption were assessed at diagnosis and recorded as binary variables. Patients were classified as NSND only if they reported never having smoked tobacco and never having consumed alcohol. Patients with any history of tobacco use or alcohol consumption, including former or occasional use, were classified as SD. This exposure-based classification was used to define an etiologically distinct OSCC phenotype lacking the two principal established carcinogenic risk factors. Pack-year history and alcohol consumption intensity (e.g., drinks per week) were not consistently documented in the retrospective record and therefore could not be analyzed as continuous exposure measures.

### 2.4. Age Stratification

Age at diagnosis was analyzed as a continuous variable and further stratified into two predefined groups: ≤50 years and >50 years. Age stratification was applied in descriptive analyses and in subgroup analyses assessing interactions between age and NSND status.

### 2.5. Clinical and Pathological Variables

Tumor subsites were categorized as tongue, mandibular gingiva, retromolar trigone, maxillary gingiva, floor of mouth, buccal mucosa, vestibule, and hard palate. Tumor stage was recorded according to the 8th edition of the AJCC/UICC TNM classification system and categorized as stage I–IVb [21]. Histopathological evaluation included tumor grade (G1–G3). Tumor burden was assessed using maximum tumor diameter and depth of invasion (DOI), both expressed in millimeters. Resection margin status was categorized as adequate (>5 mm), close, or positive. Perineural invasion (PNI), lymphovascular invasion (LVI), perivascular invasion (PVI), desmoplastic stroma, and inflammatory infiltrate were recorded as absent or present. Inflammatory infiltrate was defined as a peritumoral stromal inflammatory cell infiltrate (predominantly lymphoplasmacytic) adjacent to tumor nests and/or at the invasive front on routine H&E and was recorded dichotomously (present/absent) based on the original pathology report without formal grading. Extranodal extension (ENE) was assessed when applicable.

### 2.6. Neck Dissection and Nodal Assessment

Neck dissection status was determined based on operative records and pathology reports. Patients were classified as having undergone neck dissection if a cervical lymphadenectomy was documented in the surgical report and corresponding lymph node specimens were described in the pathology report. Nodal variables—including the total number of harvested lymph nodes, numbers of positive and negative nodes, and the presence of extranodal extension (ENE)—were analyzed exclusively in patients with a documented neck dissection. Neck dissection procedures were further classified as selective, modified radical, or radical according to the extent recorded in the operative report.

### 2.7. Immune-Related Comorbidities

Immune-modulating comorbidity was assessed as a single composite variable capturing conditions expected to alter immune function or chronic inflammatory tone at the time of OSCC diagnosis. This composite explicitly included autoimmune diseases (e.g., oral lichen planus, autoimmune thyroid disease, psoriasis, autoimmune hepatitis, vitiligo, sarcoidosis, rheumatoid arthritis, ankylosing spondylitis, and immune thrombocytopenic purpura) and other clinically relevant immune-modulating disorders (e.g., diabetes mellitus and parathyroid disorders) documented in the medical record. Diagnoses were recorded using a predefined coded list, and patients could have more than one immune-modulating diagnosis. For primary analyses, immune-modulating comorbidity was dichotomized as present/absent, and the spectrum of individual diagnoses was summarized descriptively. Throughout the manuscript, autoimmune diseases are treated as a subset of immune-modulating conditions. Diabetes mellitus was included in the immune-modulating composite as a non-autoimmune condition; only explicitly documented type 1 diabetes was considered autoimmune. Oral lichen planus was recorded only when documented prior to OSCC and confirmed histopathologically according to established oral pathology criteria. To address potential heterogeneity, we also performed sensitivity analyses restricted to autoimmune diseases only (excluding non-autoimmune metabolic/endocrine disorders such as diabetes).

### 2.8. Systemic Immune and Inflammatory Scores

Pre-treatment laboratory parameters were obtained from routine blood tests performed prior to surgical treatment. Absolute leukocyte, neutrophil, lymphocyte, monocyte, and platelet counts, as well as C-reactive protein (CRP) and serum albumin levels, were recorded when available. Based on these values, established systemic immune and inflammatory indices were calculated.

The neutrophil-to-lymphocyte ratio (NLR) was calculated as the ratio of absolute neutrophil count to absolute lymphocyte count. The platelet-to-lymphocyte ratio (PLR) was calculated as the ratio of platelet count to lymphocyte count. The lymphocyte-to-monocyte ratio (LMR) was calculated as the ratio of lymphocyte count to monocyte count. The systemic inflammatory response index (SIRI) was calculated as (neutrophils × monocytes)/lymphocytes, and the aggregate index of systemic inflammation (AISI) as (neutrophils × monocytes × platelets)/lymphocytes. The C-reactive protein-to-albumin ratio (CAR) was calculated as CRP divided by serum albumin. The prognostic nutritional index (PNI_nut) was calculated according to the following formula: 10 × serum albumin value (g/dL) + 0.005 × peripheral lymphocyte count (cells/mm^3^). All immune and inflammatory scores were analyzed as continuous variables. Because intercurrent infection and corticosteroid use can influence leukocyte-based indices, we performed a sensitivity analysis excluding patients with leukocytosis (>11 × 10^−9^/L) (Supplementary Table S3).

### 2.9. Outcomes and Follow-Up

Follow-up time was calculated in months from the date of primary surgical treatment to the date of last clinical contact or death. The primary endpoint was overall survival (OS), defined as time to death from any cause. Surviving patients were censored at last follow-up. Secondary endpoints included disease-specific survival (DSS), disease recurrence, and occurrence of second primary tumors (SPT). Patients were followed according to institutional practice in a dedicated head and neck oncology setting, typically every 1–3 months during the first 2 years, every 3–6 months during years 3–5, and annually thereafter, with clinical examination at each visit and imaging as clinically indicated. Recurrence and second primary tumor events were ascertained by review of outpatient notes, imaging reports, operative records, and pathology databases; whenever available, events were histopathologically confirmed. Follow-up time was censored at the date of last documented clinical contact for patients without an event.

### 2.10. Statistical Analysis

Statistical analyses were performed using MedCalc, version 12.5.0 (MedCalc Software, Ostend, Belgium; https://www.medcalc.org, accessed 15 November 2025). Continuous variables were summarized as medians and interquartile ranges (IQRs) and compared between groups using the Mann–Whitney U test. Comparisons across more than two groups were performed using the Kruskal–Wallis test. Categorical variables were compared using the χ^2^ test or Fisher’s exact test where appropriate. Recurrence and second primary tumor incidence were compared between exposure groups using χ^2^ test or Fisher’s exact test (two-sided), as appropriate. Age-stratified comparisons were evaluated using cross-tabulation with the χ^2^ test for interaction. Associations between clinicopathologic parameters and NSND status were assessed using univariable and multivariable logistic regression models, with results expressed as odds ratios (ORs) and 95% confidence intervals (CIs). Overall and disease-specific survival were analyzed by the Kaplan–Meier method and compared using the log-rank test. Cox proportional hazards regression was used to identify independent predictors of overall survival (OS); variables with a *p*-value < 0.10 in univariable analysis were entered into multivariable models. All *p*-values were two-sided, and *p* < 0.05 was considered statistically significant.

### 2.11. Ethical Approval

The study was approved by the Ethics Committee of Dubrava University Hospital (2024/1022-5, 22 October 2024). The requirement for written informed consent was waived due to the retrospective design and use of routinely collected clinical data. All data were anonymized prior to analysis. The study was conducted in accordance with the Declaration of Helsinki and applicable data protection regulations.

## 3. Results

### 3.1. Patient Characteristics and Cohort Overview

The final cohort comprised 243 patients with primary OSCC treated surgically between 2011 and 2020. The median age at diagnosis was 50 years (interquartile range [IQR] 46–64), and 173 patients (71.2%) were male. Median follow-up was 60 months (IQR 16–102). Systemic immune–inflammatory indices derived from pre-treatment blood tests were available for 203/243 patients (83.5%). Recurrence status was available for 241/243 patients (99.2%); two patients lacked documented recurrence information and were excluded from recurrence analyses only. Overall, 85 patients (35.0%) were classified as NSND, while 158 patients (65.0%) were classified as SD. Although a small difference in median age was observed in unadjusted analysis, age distribution was largely comparable between NSND and SD groups. In the NSND subgroup, the age distribution in our cohort suggested two age maxima, with younger patients (≤50 years; *n* = 36) clustering at 45–49 years (median 43.5 years) and older patients (>50 years; *n* = 49) clustering at 65–69 years (median 70 years). Gender distribution differed markedly between exposure groups. Female patients constituted 58.8% (50/85) of the NSND group, compared with 12.7% (20/158) in the SD group (*p* < 0.001). NSND patients also had a higher body mass index (BMI) compared with SD patients (median 24.85 vs. 23.34 kg/m^2^, *p* = 0.0019). When stratified by age, NSND status was present in both age groups, without a statistically significant difference in prevalence between patients aged ≤50 years and those >50 years (*p* = 0.073), indicating that the NSND phenotype was not confined to younger patients. Baseline demographic and clinicopathological characteristics stratified by exposure status are summarized in Table 1.

### 3.2. Tumor Subsite

**Figure 1:**
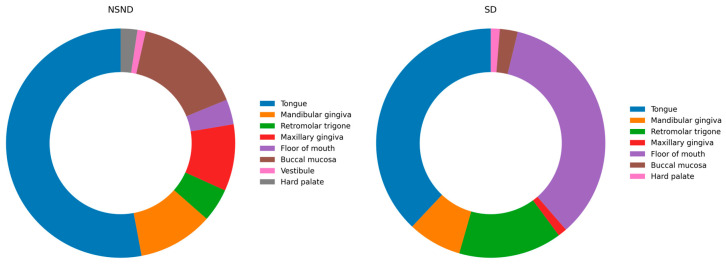
Tumor subsite distribution according to exposure status. The chart illustrates the distribution of primary OSCC subsites stratified by exposure status. In NSND patients, tumors most frequently arise in the tongue and buccal mucosa, with minimal involvement of the floor of mouth, whereas SD patients show a higher proportion of floor-of-mouth tumors, reflecting a more classical OSCC anatomical pattern. This mirror-like topographic distribution supports the concept of distinct carcinogenic pathways, with NSND tumors favoring mucosal regions subject to chronic mechanical or immunologic exposure. Tumor subsites were categorized as tongue, mandibular gingiva, retromolar trigone, maxillary gingiva, floor of mouth, buccal mucosa, vestibule, and hard palate.

Tumor anatomic distribution showed a highly significant difference between NSND and SD patients (*p* < 0.001). NSND tumors were predominantly located on the tongue (52.9%) and buccal mucosa (15.3%), whereas tumors of the floor of mouth were rare in this group (3.5%). In contrast, SD patients showed a classical OSCC distribution, with a high proportion of tumors in the floor of mouth (34.8%), followed by the tongue (38.0%) and retromolar trigone (14.6%). Tumors of the maxillary gingiva were significantly more frequent in NSND patients than in SD patients (8/85 [9.4%] vs. 2/158 [1.3%]; Fisher’s exact *p* = 0.004), corresponding to an odds ratio of approximately 8.1 (95% CI 1.68–39.08). This subsite-specific pattern persisted after age stratification, with similar NSND–SD differences observed in both ≤50 and >50 age groups; however, within the NSND subgroup, the distribution of primary tumor subsites differed significantly between patients younger than 50 years and those aged 50 years or older (χ^2^ *p* = 0.007), with younger patients showing a higher proportion of tongue and retromolar tumors and older patients exhibiting more frequent gingival involvement. This mirror-like subsite pattern suggests distinct carcinogenic pathways, with NSND tumors preferentially arising in mechanically or immunologically exposed mucosal regions. Tumor subsite distribution by exposure group is illustrated in Figure 1.

### 3.3. Tumor Burden and Histopathological Risk Features

Pathologic stage distribution (stages I–IVb) did not differ significantly between NSND and SD patients (*p* = 0.677), nor did tumor grade distribution (*p* = 0.990). However, markers of local tumor burden were significantly lower in NSND patients. Median depth of invasion (DOI) was 10 mm (IQR 5–15) in NSND patients compared with 10 mm (IQR 7–20) in SD patients (*p* = 0.036). Similarly, maximum tumor diameter was smaller in NSND patients (median 25 mm) than in SD patients (median 30 mm, *p* = 0.017). Among adverse histopathological features, perineural invasion (PNI) was significantly less frequent in NSND patients (40.5% vs. 55.1%, *p* = 0.031), and desmoplastic stroma was also less common (40.5% vs. 53.8%, *p* = 0.048). Conversely, the presence of an inflammatory infiltrate was more frequent in NSND patients (73.8% vs. 60.1%, *p* = 0.034). Rates of LVI and PVI were lower in NSND patients, although these differences did not reach statistical significance. Collectively, these findings indicate a tumor phenotype in NSND patients characterized by lower local invasiveness and a more prominent inflammatory microenvironment.

### 3.4. Neck Dissection and Nodal Disease

Neck dissection was performed less frequently in NSND patients (64.7%) compared with SD patients (79.7%, *p* = 0.010). Among patients who underwent neck dissection, the total number of harvested lymph nodes, number of positive lymph nodes, and prevalence of extranodal extension (ENE) did not differ significantly between exposure groups (all *p* > 0.70). These results indicate that the distinction between NSND and SD tumors is largely confined to local tumor behavior rather than nodal disease characteristics. Because neck dissection is influenced by stage and subsite, we performed an adjusted logistic regression including pathologic stage and tumor subsite; NSND status was not independently associated with neck dissection (adjusted OR 0.61, 95% CI 0.23–1.61; Supplementary Table S4).

### 3.5. Immune-Related Comorbidities

Immune-modulating conditions (including autoimmune diseases) were present in 85 patients (35.0%) overall. Their prevalence was markedly higher in NSND patients (67.1%) than in SD patients (17.7%, *p* < 0.001). In multivariable logistic regression adjusting for age and gender, NSND status remained independently associated with immune-modulating conditions (OR 6.25, 95% CI 3.23–12.11; *p* < 0.001). Female gender was also independently associated with immune-modulating conditions (OR 2.67, 95% CI 1.32–5.43; *p* = 0.006). The distribution of immune-modulating diagnoses by exposure group is detailed in Table 2. Multiple immune-modulating conditions were identified in 22 patients (9.1%). This multimorbidity phenotype was strongly enriched in NSND patients (20/85, 23.5%) compared with SD patients (2/158, 1.3%; Fisher’s exact *p* < 0.001). In age-stratified analysis, ≥2 conditions were observed in 7/36 (19.4%) NSND patients aged ≤50 and 13/49 (26.5%) NSND patients aged >50, whereas it remained rare in SD patients (2/86 [2.3%] in ≤50 and 0/72 in >50). Sensitivity analyses restricted to autoimmune diseases only (excluding non-autoimmune disorders such as diabetes) yielded consistent results. Autoimmune diseases were present in 53/243 patients (21.8%), including 38/85 NSND patients (44.7%) versus 15/158 SD patients (9.5%) (Fisher’s exact *p* < 0.001; adjusted OR 5.37, 95% CI 2.52–11.41). This enrichment was most pronounced in patients >50 years (25/49 [51.0%] in NSND vs. 2/72 [2.8%] in SD). (Supplementary Table S1). Oral lichen planus—histologically confirmed prior to OSCC—was documented in 18/85 NSND patients (21.2%) and 2/158 SD patients (1.3%) (*p* < 0.001).

### 3.6. Age-Stratified Immune Phenotype

**Figure 2:**
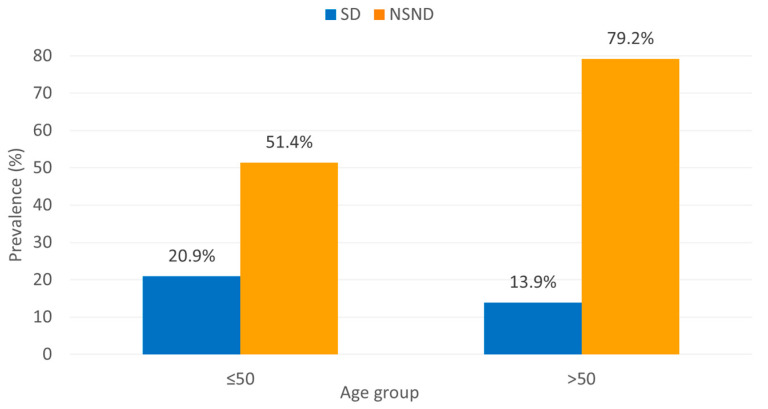
Prevalence of immune-modulating conditions stratified by age and exposure status. The bar chart illustrates the proportion of patients with immune-modulating conditions (including autoimmune diseases) across age groups (≤50 years vs. >50 years) and exposure categories (NSND vs. SD). A pronounced enrichment of immune-modulating conditions was observed in older NSND patients (χ^2^ = 7.232, *p* = 0.007), while the SD group showed no significant age-related variation.

Age-stratified analyses demonstrated that immune-modulating conditions were particularly enriched in older NSND patients (>50 years). Immune-modulating conditions were present in 79.2% of NSND patients aged >50 years compared with 51.4% of those aged ≤50 years (χ^2^ = 7.232, *p* = 0.007). In contrast, within the SD group, the prevalence of immune-modulating conditions did not increase with age (13.9% in >50 vs. 20.9% in ≤50 years). These results are summarized in Table 3 and illustrated in Figure 2.

### 3.7. Systemic Immune and Inflammatory Scores

**Figure 3:**
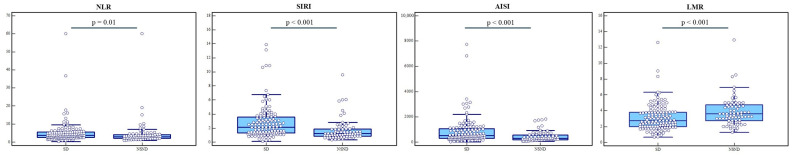
Systemic immune and inflammatory biomarkers according to exposure status. Box-and-whisker plots with individual data points illustrating the distribution of neutrophil-to-lymphocyte ratio (NLR), systemic inflammatory response index (SIRI), aggregate index of systemic inflammation (AISI), and lymphocyte-to-monocyte ratio (LMR) in SD and NSND patients. Boxes represent median and interquartile range, with whiskers indicating 1.5× IQR. Group comparisons were performed using the Mann–Whitney U test, with *p*-values displayed above each SD–NSND comparison. The dots represent individual patient values.

Systemic immune and inflammatory indices differed significantly between exposure groups. NSND patients exhibited lower levels of systemic inflammation, with significantly lower neutrophil-to-lymphocyte ratio (NLR) (*p* = 0.01), systemic inflammatory response index (SIRI) (*p* < 0.001), and aggregate index of systemic inflammation (AISI) (*p* < 0.001), alongside a higher lymphocyte-to-monocyte ratio (LMR) (*p* < 0.001). PLR, dNLR, CAR, and PNI_nut did not differ significantly between groups. These findings indicate that, despite a higher prevalence of immune-related comorbidities, NSND patients demonstrate a less pronounced systemic inflammatory response at diagnosis. In exploratory analyses stratifying the SD group by exposure type, systemic inflammatory indices showed a stepwise pattern across three exposure categories (NSND vs. smoking-only vs. smoking + alcohol), with the highest inflammatory indices in patients with combined smoking and alcohol exposure (Supplementary Table S5). Comparisons of systemic immune scores are shown in Figure 3.

In a sensitivity analysis excluding patients with leukocytosis (>11 × 10^−9^/L), NSND patients continued to show lower NLR (*p* = 0.048), lower SIRI (*p* < 0.001) and AISI (*p* = 0.002), and higher LMR (*p* = 0.004) (Supplementary Table S3).

### 3.8. Survival Outcomes

**Figure 4:**
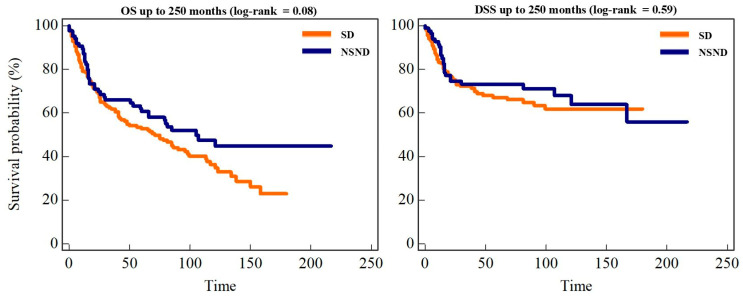
Overall and disease-specific survival according to exposure status. Kaplan–Meier curves for overall survival (OS) and disease-specific survival (DSS) according to exposure status (NSND vs. SD). Curves are shown up to 250 months, while log-rank *p*-values were calculated on the full follow-up; OS demonstrated a non-significant trend toward improved survival in NSND patients (*p* = 0.08), whereas DSS did not differ significantly between groups (*p* = 0.59).

During follow-up, 138 deaths (56.8%) occurred. Median overall survival (OS) for the entire cohort was 81.2 months. Estimated OS rates were 82.3% at 12 months, 70.1% at 24 months, 55.9% at 60 months, and 40.1% at 120 months. Median OS was longer in NSND patients (105.8 months) than in SD patients (71.2 months), with a trend toward improved survival (log-rank *p* = 0.083). Five-year OS was 60.6% in NSND patients versus 53.4% in SD patients. In Cox regression analysis, NSND status was associated with improved OS after adjustment for age and gender (HR 0.64, 95% CI 0.42–0.98; *p* = 0.041). However, this association was attenuated after further adjustment for tumor stage, DOI, PNI, LVI, and resection margin status, indicating that tumor-related characteristics largely accounted for the observed survival difference (Table 4). Disease-specific survival (DSS) did not differ significantly between NSND and SD patients (*p* = 0.59). Kaplan–Meier survival curves are shown in Figure 4.

**Figure 5:**
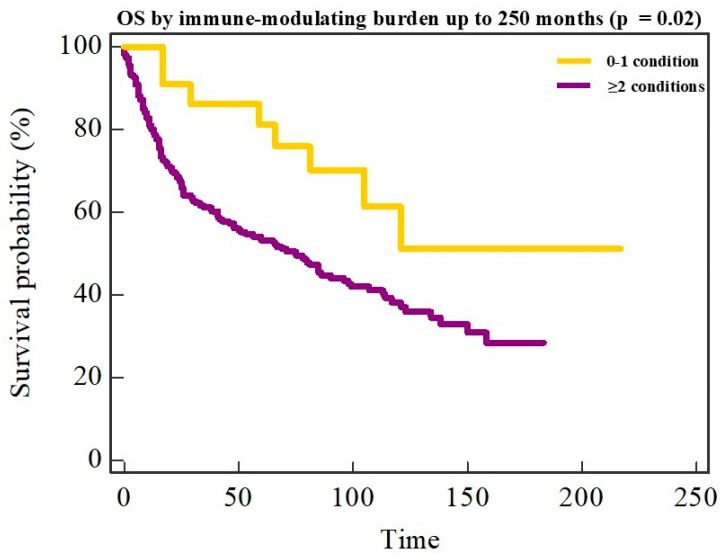
Overall survival according to immune-modulating comorbidity burden. Kaplan–Meier curves for overall survival (OS) according to the number of immune-modulating conditions (0–1 vs. ≥2). Patients with ≥2 immune-modulating conditions demonstrated significantly longer OS (log-rank *p* = 0.02). Survival curves are shown up to 250 months of follow-up.

Kaplan–Meier analysis further demonstrated significantly improved OS in patients with multiple immune-modulating conditions compared with those with zero or one condition (log-rank *p* = 0.02). Median OS was not reached in the multiple-condition group, whereas it was approximately 75 months in patients with ≤1 immune-modulating condition (Figure 5).

### 3.9. Recurrence and Second Primary Tumors

Disease recurrence occurred in 83 of 241 evaluable patients (34.2%), with no significant difference between NSND and SD groups (*p* = 0.82). Two patients were excluded from recurrence analyses because recurrence status was not documented in the retrospective record. Second primary tumors were identified in 82 patients (33.7%), most frequently within the head and neck region, followed by lung malignancies. The incidence and anatomical distribution of second primary tumors did not differ between exposure groups.

## 4. Discussion

Our findings support the concept that NSND OSCC represents a distinct clinicobiological phenotype rather than simply “OSCC without exposure.” In our surgically treated cohort, 35% of patients met strict NSND criteria, consistent with recent reports indicating that NSND cases account for approximately 20–35% of OSCC, depending on definitions and cohort composition [5,8,22]. NSND patients were, on average, older at diagnosis and showed a marked female predominance (58.8% in NSND Vs. 12.7% in SD). In our series, the phenotype was further characterized by a “mirror-like” subsite redistribution, with NSND tumors predominantly arising in the tongue (52.9%) and buccal mucosa (15.3%) and being rare in the floor of the mouth (3.5%), whereas SD patients exhibited the classical predominance of floor of mouth tumors. This non-classical subsite pattern aligns with an inflammation-associated oral carcinogenesis framework. Meta-analytic data on oral lichen planus and related lesions, encompassing more than 26,000 patients, indicate that malignant transformation occurs most frequently on the tongue (about one third of OSCC cases), with a higher risk compared with other oral subsites (RR = 1.82), followed by buccal and gingival sites; in contrast, floor-of-mouth involvement consistently remains below 5–10% [23,24]. Epidemiologic data in older adults show that roughly 75% of individuals aged 65 years or older exhibit at least one oral mucosal lesion, and approximately 12–13% present with denture-related inflammatory changes, supporting a high background prevalence of chronic mucosal irritation in the age group where NSND tumors were enriched in our cohort [25]. Periodontal inflammation provides an additional mechanistic rationale: severe periodontitis affects around 10% of adults and may expose an ulcerated periodontal surface area of up to 8–20 cm^2^, creating a sustained pro-inflammatory microenvironment driven by dysbiotic microbiota and cytokine signaling [26]. Together, these quantitative observations support the plausibility of a non–carcinogen-driven pathway of oral carcinogenesis that preferentially affects tongue and buccal/gingival subsites.

The main contribution of our study is the significant enrichment of immune-modulating conditions in NSND OSCC, especially among patients over 50 years old. We defined immune-modulating conditions as a composite clinical variable that includes autoimmune diseases and other disorders known to alter immune function or chronic inflammatory tone. These conditions were present in 67.1% of NSND patients compared with 17.7% of SD patients, with NSND status remaining independently associated with immune-modulating comorbidity after adjustment for age and gender. These clinical observations complement previous work suggesting that NSND OSCC is associated with a distinct immune microenvironment and immune checkpoint–related signaling, including interferon-γ–related pathways and PD-1/PD-L1 and IDO1 biology in HPV-negative NSND disease [16,26]. Although our study did not include direct immune phenotyping, the co-occurrence of a high immune-modulating comorbidity burden with histologic and systemic inflammatory patterns supports the interpretation that NSND status reflects a clinically meaningful host–tumor context rather than a purely exposure-defined residual category. At the tumor level, NSND cancers in our cohort showed features consistent with reduced local aggressiveness (lower depth of invasion and less frequent perineural invasion) and more frequent histologic inflammatory infiltrates. This aligns with recent histopathologic frameworks that distinguish stromal “inflammatory” from “fibrotic/desmoplastic” patterns: peritumoral stromal inflammation (PTSI) has been reported to be more common in women and NSND patients and is associated with smaller tumor size, whereas fibrotic/desmoplastic stromal patterns (PTSF) are more frequent in males, smokers, and alcohol consumers and correlate with larger tumors and more advanced stage [27]. Immunohistochemical studies further support an immune-involved context in NSND OSCC, reporting higher infiltration of CD4^+^ and CD8^+^ T lymphocytes, higher overall tumor-infiltrating lymphocyte burden, and lower perineural invasion frequency compared with smoking- and drinking-associated disease, along with more favorable OS and DSS in some cohorts [28]. Overall, these data support the view that NSND tumors more often arise in an inflammatory or immune-conditioned mucosal field and may display distinct stromal behavior compared with classical carcinogen-driven OSCC.

At the systemic level, NSND patients in our cohort showed lower tumor-associated systemic inflammatory indices (lower NLR, SIRI, and AISI, and higher LMR; all *p* ≤ 0.01), indicating a less pronounced systemic inflammatory response at diagnosis. This finding is consistent with evidence that elevated systemic inflammatory indices primarily reflect tumor burden and advanced disease, rather than comorbidity-driven immune modulation. In a meta-analysis of more than 4500 oral cancer patients, high pretreatment SII and SIRI were associated with worse overall survival (pooled HR 1.62 and 1.60, respectively) [18]. Integrative analyses of the tumor macro- and microenvironment further support that higher systemic inflammatory ratios are linked to adverse tumor biology and poorer outcomes across squamous cell carcinomas [29]. In OSCC specifically, clinicopathologic studies report that elevated NLR and SII are independently associated with worse overall survival (multivariate HR for NLR approximately 1.6), and that lymphocyte-favoring ratios (e.g., higher LMR) correlate with a more lymphocyte-rich local immune context [17]. Within this framework, the lower systemic inflammatory indices observed in NSND patients likely reflect the less invasive tumor phenotype in our cohort, rather than an absence of immune involvement.

Field cancerization remains clinically relevant in surgically treated OSCC. In our cohort, recurrence occurred in 34.2% (83/241 evaluable patients) and second primary tumors in 33.7% (82/243), with no differences by exposure phenotype. This finding contrasts with recent prospective data suggesting an increased risk of metachronous second primary tumors in NSND patients (adjusted HR ~3.9), interpreted as reflecting field mechanisms independent of tobacco and alcohol and potentially linked to chronic mucosal inflammation and OPMD [30]. Because the broader literature remains heterogeneous and largely retrospective, with variability in phenotype definitions, missing data, and subsite composition, prospective studies with standardized NSND criteria and systematic documentation of OPMD and immunomodulatory treatments are needed to clarify long-term field vulnerability [31,32].

The etiology of NSND OSCC is likely multifactorial. Viral hypotheses remain of interest; however, methodologically rigorous studies focusing specifically on NSND OSCC indicate that high-risk Alphapapillomavirus is rare and typically transcriptionally inactive in oral cavity tumors. High-risk HPV DNA was detected in only 4.4% of NSND OSCC cases, with viral transcripts identified in a single immunocompromised patient, while Betapapillomavirus DNA was frequently detected without transcriptional activity, supporting incidental presence rather than causal oncogenesis [5,33]. Our study did not include systematic p16 immunohistochemistry or viral testing, which limits etiologic inference at the individual level. However, this limitation should be considered in the context of broader evidence suggesting limited HPV relevance in OSCC compared with oropharyngeal cancer.

Survival outcomes in NSND OSCC remain inconsistent across published series. In our cohort, NSND status showed a modest unadjusted trend toward improved OS that diminished after adjustment for tumor stage and adverse pathological features (DOI, PNI, LVI and resection margin status), consistent with large institutional data showing no independent survival benefit after multivariable adjustment in NSND OSCC [7]. Prospective evidence suggests that etiologic phenotype may be more strongly linked to long-term field risk than to primary tumor lethality [30]. Immunophenotyping studies provide partial context: while PD-L1 expression alone is not consistently prognostic, CD8^+^ TIL density has been associated with disease-free survival, and low CD8^+^ TIL density correlates with adverse features such as perineural invasion, reinforcing the prognostic importance of established pathological risk factors [34]. Interestingly, patients with multiple immune-modulating conditions demonstrated significantly improved overall survival compared with those with zero or one such condition. This finding should not be interpreted as a protective effect of comorbidity per se, but rather as a marker of a distinct host–tumor immune context. In our cohort, multiple immune-modulating conditions clustered with features of an immune-active phenotype, including increased histologic inflammation, lower DOI, reduced PNI, and a lymphocyte-favoring systemic inflammatory profile, all of which are associated with less aggressive tumor behavior. These data support the concept that immune-modulating multimorbidity identifies a biologically distinct subgroup in which tumor evolution and host response differ from classical carcinogen-driven OSCC.

Several limitations should be acknowledged. First, the retrospective single-center design introduces selection and information biases, including potential misclassification of smoking and alcohol exposure when intensity and duration data are unavailable. Additionally, pack-year and weekly alcohol intake data were not consistently available, precluding dose–response analyses between exposure intensity and systemic immune–inflammatory indices. Second, immune-modulating conditions were coded from real-world documentation and intentionally defined broadly to include autoimmune diseases within the composite; while clinically pragmatic, this approach introduces within-group heterogeneity and does not capture comorbidity severity or the effect of concomitant immunomodulatory therapies. Third, systemic immune-inflammatory indices can be influenced by intercurrent infection, medications (including steroids or other immunomodulators), nutritional status, and timing relative to surgery; although these indices were analyzed as supportive biomarkers, residual confounding is inevitable and missingness may limit generalizability. Fourth, we lacked standardized data on OPMD beyond coded diagnoses, HPV and other viral markers, microbiome features, and direct measures of the tumor immune microenvironment (e.g., tumor-infiltrating lymphocyte subsets or checkpoint expression). In addition, histologic inflammatory infiltrate was captured as a binary variable from routine pathology reports without centralized re-review, and formal inter-observer variability assessment was not performed. Future prospective studies integrating these layers are needed to validate the proposed phenotype and to clarify mechanisms.

Despite these constraints, the integrated pattern in our cohort—female predominance, subsite redistribution away from the floor of the mouth, markedly higher immune-modulating burden (including autoimmune diseases) especially in older NSND patients, increased histologic inflammation with lower invasive pathological features, and distinct systemic inflammatory signatures—supports a clinically useful reframing: NSND OSCC should be considered a positive phenotype with immune-related clinical correlates, not a residual category defined by missing exposures. Clinicians should maintain a high index of suspicion for OSCC in NSND patients with chronic oral inflammatory disease or other immune-modulating conditions, particularly older women, and follow-up strategies should emphasize careful whole-mucosa assessment and long-term surveillance given the overall burden of recurrence and second primary tumors. At the research level, NSND cohorts are strong candidates for integrated immune-microbiome-viral studies and for evaluating whether immune checkpoint biology or immunopreventive strategies have differential relevance in etiologically distinct oral cavity cancer phenotypes.

## 5. Conclusions

Non-smoking, non-drinking oral squamous cell carcinoma demonstrates a distinct clinicopathologic profile, characterized by female predominance, a non-classical anatomic distribution, lower local tumor aggressiveness, and more frequent histologic inflammatory infiltrates. NSND status is strongly associated with immune-modulating comorbidity and with lower levels of established systemic inflammatory indices used as surrogate markers of tumor-associated systemic inflammatory response, particularly in older patients. Although NSND status was not an independent predictor of survival after full clinicopathologic adjustment, it identifies a clinically relevant subgroup with features consistent with an immune-modulated disease context. Further prospective studies integrating standardized immune phenotyping are warranted to clarify the biological mechanisms underlying these associations and their implications for risk stratification and surveillance.

## Figures and Tables

**Table 1:** Baseline Demographic and Clinicopathological Characteristics of the Cohort Stratified by Exposure Status.

Variable	Total (*N* = 243)	NSND (*n* = 85)	SD (*n* = 158)	*p*-Value
Age, years	50 (46–64)	54 (48–66)	50 (45–63)	0.036
Female sex	70 (28.8)	50 (58.8)	20 (12.7)	<0.001
BMI, kg/m^2^	24.1 (21.0–27.0)	24.85 (22.1–27.9)	23.34 (20.8–26.1)	0.0019
Tumor subsite				<0.001
–Tongue	105 (43.2)	45 (52.9)	60 (38.0)	
–Floor of mouth	58 (23.9)	3 (3.5)	55 (34.8)	
–Buccal mucosa	17 (7.0)	13 (15.3)	4 (2.5)	
–Mandibular gingiva	21 (8.6)	9 (10.6)	12 (7.6)	
–Maxillary gingiva	10 (4.1)	8 (9.4)	2 (1.3)	
–Retromolar trigone	27 (11.1)	4 (4.7)	23 (14.6)	
–Vestibule	1 (0.4)	1 (1.2)	0 (0.0)	
–Hard palate	4 (1.6)	2 (2.4)	2 (1.3)	
Pathologic stage				0.677
–I–II	69 (28.4)	26 (30.6)	43 (27.4)	
–III–IVb	174 (71.6)	59 (69.4)	115 (72.8)	
Tumor grade (G3)				0.990
–G1	135 (55.6)	47 (55.3)	88 (55.7)	
–G2	86 (35.4)	30 (35.3)	56 (35.4)	
–G3	22 (9.1)	8 (9.4)	14 (8.9)	
DOI, mm	10 (6–20)	10 (5–15)	10 (7–20)	0.036
Tumor size, mm	30 (20–36)	25 (15–35)	30 (20–39)	0.017
PNI present	121 (50.0)	34 (40.5)	87 (55.1)	0.031
Desmoplastic stroma	119 (49.0)	34 (40.5)	85 (53.8)	0.048
Inflammatory infiltrate	157 (64.9)	62 (73.8)	95 (60.1)	0.034
Neck dissection	181 (74.5)	55 (64.7)	126 (79.7)	0.010

Abbreviations: NSND, non-smoking non-drinking; SD, smoking and/or drinking; BMI, body mass index; DOI, depth of invasion; PNI, perineural invasion.

**Table 2:** Spectrum of Immune-Modulating Conditions (autoimmune diseases and non-autoimmune immune-modulating disorders).

Immune-Modulating Conditions	NSND (*n* = 57)	SD (*n* = 28)
Autoimmune diseases		
1. Diabetes mellitus type 1	10 (17.5%)	5 (17.9%)
2. Oral lichen planus	18 (31.6%)	2 (7.1%)
3. Hashimoto thyroiditis	13 (22.8%)	1 (3.6%)
4. Psoriasis	2 (3.5%)	4 (14.3%)
5. Immune thrombocytopenic purpura	3 (5.3%)	0 (0.0%)
6. Vitiligo	1 (1.8%)	2 (7.1%)
7. Graves’ disease	2 (3.5%)	0 (0.0%)
8. Rheumatoid arthritis	1 (1.8%)	1 (3.6%)
9. Autoimmune Polyglandular Syndrome type 1	2 (3.5%)	0 (0.0%)
10. Autoimmune hepatitis	1 (1.8%)	0 (0.0%)
11. Ankylosing spondylitis	1 (1.8%)	0 (0.0%)
12. Multiple sclerosis	1 (1.8%)	0 (0.0%)
13. Dermatitis herpetiformis (celiac-related)	1 (1.8%)	0 (0.0%)
14. Alopecia areata	1 (1.8%)	1 (3.6%)
15. Sarcoidosis	1 (1.8%)	0 (0.0%)
Non-autoimmune disorders		
1. Diabetes mellitus type 2/unspecified	13 (22.8%)	7 (25.0%)
2. Multiple myeloma	0 (0.0%)	2 (7.1%)
3. Organ transplantation	0 (0.0%)	1 (3.6%)
4. Leukemia	2 (3.5%)	0 (0.0%)
5. Myeloproliferative neoplasia	1 (1.8%)	1 (3.6%)
6. Viral hepatitis	1 (1.8%)	1 (3.6%)
7. Thrombophilia	1 (1.8%)	1 (3.6%)
8. Hyperparathyroidism	1 (1.8%)	0 (0.0%)
9. Severe allergies	2 (3.5%)	2 (7.1%)

**Table 3:** Age-stratified comparison of NSND and SD phenotypes.

Variable	≤50 NSND (*n* = 36)	≤50 SD (*n* = 86)	>50 NSND (*n* = 49)	>50 SD (*n* = 72)	*p* (Interaction)
Female sex, *n* (%)	19 (51.4)	9 (10.5)	31 (64.6)	11 (15.3)	<0.001
Immune-modulating condition	19 (51.4)	18 (20.9)	38 (79.2)	10 (13.9)	0.007
Autoimmune disease (only)	13 (36.1)	13 (15.1)	25 (51.0)	2 (2.8)	0.007
DOI, mm	9 (4–14)	11 (7–20)	10 (6–15)	12 (8–22)	0.041

**Table 4:** Survival Outcomes and Cox Regression Analysis. Cox proportional hazards model for overall survival (OS) according to exposure status and key clinicopathologic variables.

Variable	HR (95% CI)	*p*-Value
NSND (unadjusted)	0.72 (0.50–1.04)	0.083
NSND + age + sex	0.64 (0.42–0.98)	0.041
NSND + clinicopathologic factors	0.76 (0.54–1.27)	0.231
Stage IV	2.91 (1.96–4.33)	<0.001
DOI (per mm)	1.03 (1.01–1.05)	0.004
PNI	1.68 (1.17–2.41)	0.005

## Data Availability

All data generated or analyzed during this study are included in this published article.

## Supplementary Materials

The following supporting information can be downloaded at: https://www.mdpi.com/article/10.3390/cancers18040553/s1, Figure S1: STROBE-style flow diagram of cohort assembly and analytic datasets. Table S1: Autoimmune diseases-only sensitivity analysis: prevalence by exposure status and age strata. Table S2: Multivariable logistic regression for autoimmune disease presence (autoimmune diseases only): association with NSND status adjusted for age and gender. Table S3: Sensitivity analysis excluding leukocytosis (WBC > 11 × 10^9^/L): systemic inflammatory indices by exposure status. Table S4: Multivariable logistic regression for neck dissection: association with exposure status adjusted for tumor subsite, stage, age, and gender. Table S5: Systemic immune–inflammatory indices stratified by exposure type (NSND vs. smoking-only vs. smoking + alcohol).

## Abbreviations

The following abbreviations are used in this manuscript:

AISI	Aggregate Index of Systemic Inflammation
AJCC	American Joint Committee on Cancer
BMI	Body Mass Index
CAR	C-reactive Protein–to–Albumin Ratio
CRP	C-reactive Protein
DOI	Depth of Invasion
DSS	Disease-Specific Survival
ENE	Extranodal Extension
HPV	Human Papillomavirus
LMR	Lymphocyte-to-Monocyte Ratio
LVI	Lymphovascular Invasion
NLR	Neutrophil-to-Lymphocyte Ratio
NSND	Non-Smoking, Non-Drinking
OPMD	Oral Potentially Malignant Disorders
OPSCC	Oropharyngeal Squamous Cell Carcinoma
OSCC	Oral Squamous Cell Carcinoma
PLR	Platelet-to-Lymphocyte Ratio
PNI	Perineural Invasion
PNI_nut	Prognostic Nutritional Index
PVI	Perivascular Invasion
SD	Smoking and/or Drinking
SII	Systemic Immune-Inflammation Index
SIRI	Systemic Inflammation Response Index
SPT	Second Primary Tumor
TILs	Tumor-Infiltrating Lymphocytes
TNM	Tumor–Node–Metastasis
UICC	Union for International Cancer Control
