# Supplementary material for: Non-Smoking, Non-Drinking Oral Squamous Cell Carcinoma Is Associated with an Immune-Modulated Clinical Phenotype
Source: Cancers (Basel). 2026 Feb 8;18(4):553. doi: 10.3390/cancers18040553 (PMC12938323; doi:10.3390/cancers18040553)
Supplement: Supplementary file 1 [file cancers-18-00553-s001.zip › cancers-4117414-supplementary.pdf]

Supplementary Figures

Figure S1. STROBE-style flow diagram of cohort assembly and analytic datasets.

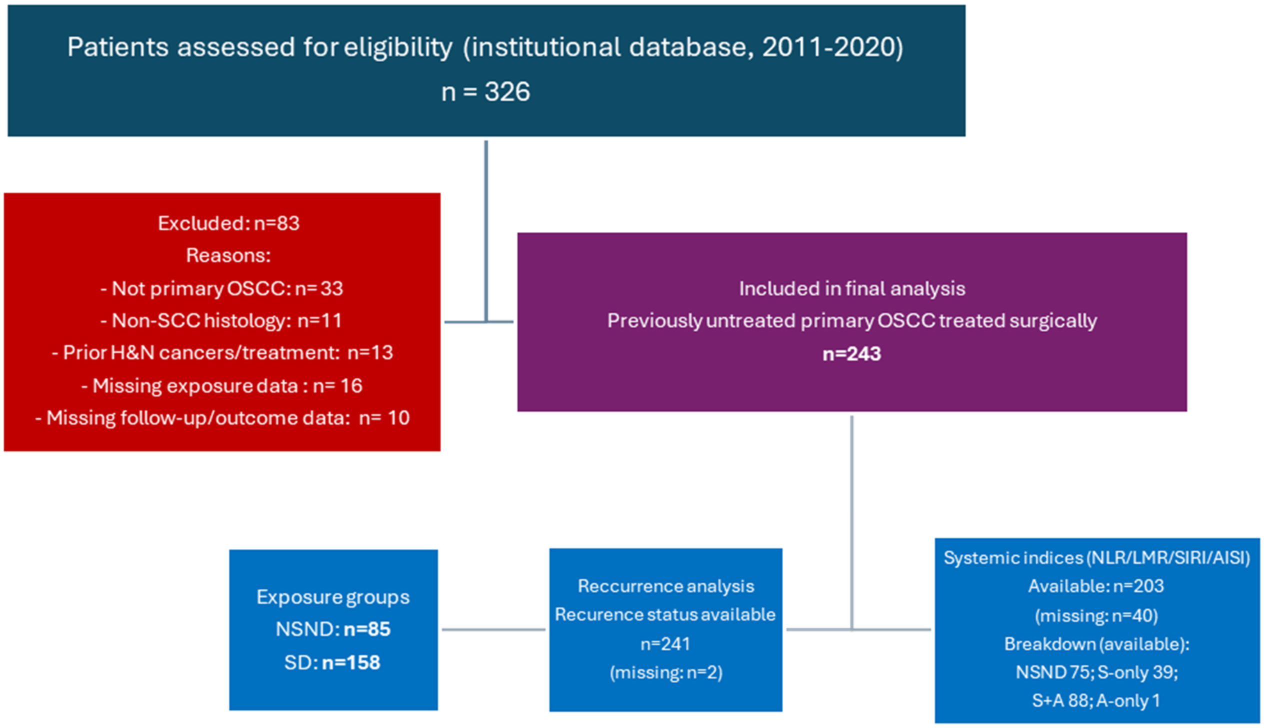

Abbreviations: OSCC, oral squamous cell carcinoma; SCC, squamous cell carcinoma; H&N, head and neck; NSND, non-smoker/non-drinker; SD, smoker and/or drinker; NLR, neutrophil-to-lymphocyte ratio; SIRI, systemic inflammatory response index; AISI, aggregate index of systemic inflammation; LMR, lymphocyte-to-monocyte ratio.

Supplementary Tables

Table S1. Autoimmune diseases-only sensitivity analysis: prevalence by exposure status and age strata.

Stratum	Autoimmune NSND n	NSND N	Autoimmune SD n	SD N	OR	CI low	CI high	p
**Overall**	38	85	15	158	7.71	3.89	15.25	<0.001
**Age ≤50**	13	36	13	86	3.17	1.29	7.81	0.015
**Age >50**	25	49	2	72	36.46	8.03	165.54	<0.001

Abbreviations: NSND, non-smoker/non-drinker; SD, smoker and/or drinker. Notes: Odds ratios (ORs) and 95% confidence intervals (CIs) were calculated from 2×2 contingency tables. P-values were obtained using Fisher's exact test (two-sided).

Table S2. Multivariable logistic regression for autoimmune disease presence (autoimmune diseases only): association with NSND status adjusted for age and gender.

Predictor	Adjusted OR	95% CI low	95% CI high	p
**NSND vs SD**	5.37	2.52	11.41	<0.001
**Female gender**	2.32	1.09	4.98	0.029
**Age (per year)**	0.99	0.98	1.02	0.96

Abbreviations: NSND, non-smoker/non-drinker; SD, smoker and/or drinker. Adjusted odds ratios (ORs) with 95% CIs were estimated using multivariable logistic regression. p-values are based on the Wald test.

Table S3. Sensitivity analysis excluding leukocytosis (WBC > 11 × 10^9/L): systemic inflammatory indices by exposure status.

Index	NSND median (IQR)	SD median (IQR)	p
**NLR**	3.00 (2.11–3.72)	3.35 (2.46–4.93)	0.048
**LMR**	3.60 (3.00–4.63)	3.00 (2.10–4.00)	0.0038
**SIRI**	1.24 (0.86–1.83)	1.61 (1.20–2.90)	<0.001
**AISI**	296.01 (200.11–467.78)	433.52 (284.08–824.24)	0.0015

Abbreviations: NSND, non-smoker/non-drinker; SD, smoker and/or drinker; NLR, neutrophil-to-lymphocyte ratio; SIRI, systemic inflammatory response index; AISI, aggregate index of systemic inflammation; LMR, lymphocyte-to-monocyte ratio. Notes: Data are presented as median (IQR). Group comparisons were performed using the Mann–Whitney U test (two-sided).

Table S4. Multivariable logistic regression for neck dissection: association with exposure status adjusted for tumor subsite, stage, age, and gender.

Predictor	Adjusted OR	95% CI (low)	95% CI (high)	p
**Mandibular gingiva vs Tongue**	2.47	0.35	17.58	0.37
**Retromolar trigone vs Tongue**	2.64	0.56	12.40	0.22
**Maxillary gingiva vs Tongue**	0.06	0.01	0.27	<0.001
**Floor of mouth vs Tongue**	0.64	0.22	1.87	0.42
**Buccal mucosa vs Tongue**	1.96	0.45	8.59	0.37
**Hard palate vs Tongue**	0.069	0.01	0.61	0.016
**NSND vs SD**	0.61	0.23	1.61	0.32
**Stage III–IVb vs I–II**	31.90	13.06	77.92	<0.001
**Age (per year)**	1.00	0.97	1.03	0.93
**Female gender**	0.59	0.24	1.47	0.26

Notes: Adjusted odds ratios (ORs) with 95% CIs were estimated using multivariable logistic regression. P-values are based on the Wald test.

Table S5. Systemic immune–inflammatory indices stratified by exposure type (NSND vs smoking-only vs smoking+alcohol).

Index	NSND (n)	NSND median (IQR)	S-only (n)	S-only median (IQR)	S+A (n)	S+A median (IQR)	p
**NLR**	75	3.05 (2.15–4.06)	39	3.30 (2.46–5.53)	88	3.79 (2.83–5.35)	0.034
**LMR**	75	3.60 (2.77–4.71)	39	3.20 (1.89–3.92)	88	2.60 (2.00–3.76)	0.002
**SIRI**	75	1.24 (0.86–1.84)	39	1.90 (1.06–3.44)	88	2.26 (1.38–3.48)	<0.001
**AISI**	75	309.78 (200.11–532.87)	39	489.94 (243.04–1035.71)	88	512.34 (303.62–1045.76)	<0.001
**PLR**	75	165.56 (123.12–227.79)	39	156.25 (108.03–239.17)	88	168.14 (125.14–255.62)	0.777
**CAR**	74	0.01 (0.00–0.12)	41	0.02 (0.00–0.22)	84	0.00 (0.00–0.28)	0.811
**PNI_nut**	85	49.50 (44.00–53.50)	53	48.50 (33.50–55.00)	104	48.00 (37.62–53.00)	0.409

Abbreviations: NSND, never-smoker/never-drinker; S-only, smoking only; S+A, smoking plus alcohol; NLR, neutrophil-to-lymphocyte ratio; LMR, lymphocyte-to-monocyte ratio; SIRI, systemic inflammatory response index; AISI, aggregate index of systemic inflammation; PLR, platelet-to-lymphocyte ratio; CAR, C-reactive protein-to-albumin ratio; PNI_nut, prognostic nutritional index; IQR, interquartile range. Notes: Data are presented as median (IQR). Overall group differences across the three exposure categories were assessed using the Kruskal–Wallis test (two-sided).
